# Diagnostic Dilemma: Parasitic Ovarian Fibroma With Degeneration

**DOI:** 10.7759/cureus.32739

**Published:** 2022-12-20

**Authors:** Sweta Singh, Pooja Sahu, Advika T Selvan, Suprava Naik

**Affiliations:** 1 Obstetrics and Gynecology, All India Institute of Medical Sciences, Bhubaneswar, Bhubaneswar, IND; 2 Obstetrics and Gynaecology, All India Institute of Medical Sciences, Bhubaneswar, Bhubaneswar, IND; 3 Radiodiagnosis, All India Institute of Medical Sciences, Bhubaneswar, Bhubaneswar, IND

**Keywords:** parasitic, gastrointestinal stromal tumor, fibroma, fibroid, case report

## Abstract

An ovarian fibroma is a rare entity and a diagnostic dilemma due to its solid nature and ultrasound findings being similar to a uterine fibroid. An ovarian fibroma, being parasitic, is extremely rare. We report the case of a 35-year-old, multiparous woman who presented with a ‘wandering’ abdominal mass of six months duration. Clinical examination revealed a 16-week size, solid, firm, well-defined mass in the right lumbar and iliac regions, separate from the uterus on bimanual examination, suggestive of a pedunculated subserous fibroid uterus. Ultrasound abdomen with color Doppler evaluation was suggestive of the non-uterine origin of the tumor, probably a gastrointestinal stromal tumor. In view of the diagnostic dilemma, MRI was done, which showed that the lesion had features characteristic of ovarian fibroma, however, bilateral ovaries were normally visualized. An exploratory laparotomy was performed. There was a solid parasitic tumor adherent to the bladder peritoneum and attached to the right ovary by a thin band, which was excised. Histopathology confirmed ovarian fibroma. To conclude, a parasitic ovarian fibroma is rare and a careful clinical approach with imaging and surgery helps in solving this diagnostic dilemma.

## Introduction

Ovarian fibroma is an uncommon sex cord-stromal tumor, accounting for around 1-4% of all ovarian tumors [1]. It commonly affects women in the fifth decade of life and is often misdiagnosed preoperatively as uterine fibroid due to its solid nature and similar clinical and ultrasound (USG) findings. Surgical cause like a large gastrointestinal stromal tumor (GIST) is also a strong differential diagnosis [2]. Ovarian fibroma being parasitic is extremely rare [3]. Here, we present the case of a woman in her thirties with parasitic ovarian fibroma with degeneration and discuss the diagnostic challenges. This article was previously posted to the Research Square preprint server on September 16, 2022.

## Case presentation

A 35-year-old multiparous woman presented with an abdominal mass of six months duration. The mass was gradually increasing in size for the last four months and in her words, was ‘wandering’ in her abdomen. It was also associated with mild abdominal pain. Her menstrual cycles were regular, with average flow and mild dysmenorrhoea. Her last menstrual period was eight days back. She was multiparous, with previous two cesarean deliveries and her last childbirth was seven years ago. She was not on any contraceptives. There was no history of loss of appetite or recent weight loss. Her past history and family history were unremarkable.

On general examination, she was of average build and nutrition. There was no pallor, and her vitals were stable. Abdominal examination revealed a firm, solid mass corresponding to 16 weeks of gestation. It was felt on the right lumbar and iliac regions and was mobile from side to side. Its lower edge was not palpable. On bimanual examination, the mass was felt separate from the uterus in the right fornix, measured approximately 10x4 cm, and was mobile. The left fornix was free. With a provisional diagnosis of pedunculated subserous fibroid uterus, she was worked up further.

**Figure 1:**
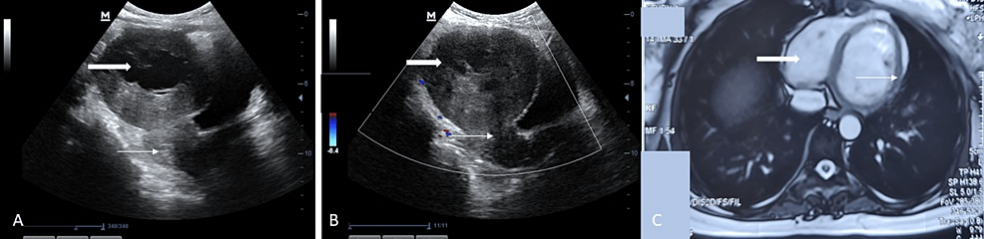
Pre-operative imaging A. 2D ultrasound showing a normal-sized uterus (thin arrow) with a solid lesion of size 8x7 cm with central degeneration (thick arrow) present superior to the uterus, suggestive of subserous uterine fibroid B. Color Doppler ultrasound showing the large hypoechoic solid mass with anechoic spaces and no internal vascularity (thick arrow). The bridging vascular sign with the uterus was absent (thin arrow), suggestive of the non-uterine origin of the mass, presumably a large gastrointestinal stromal tumor. C. MRI showing T2 hyperintensity suggestive of cystic degeneration (thick arrow), with a band of T2 hypointensity (thin arrow) separating the tumor from the uterus on all planes, characteristic of an ovarian fibroma.

Transabdominal USG revealed a normal-sized uterus with thin endometrium. There was the presence of a large, lobulated, hypoechoic mass lesion in the abdominal cavity, measuring 9.6x8.4x7.9cm, with an anechoic center, which was superior to the uterus and indented the urinary bladder (Figure 1A).

On color Doppler USG, there was no internal vascularity present in the lesion. The bridging vascular sign with the uterus was absent, suggestive of the non-uterine origin of the tumor, probably a GIST (Figure 1B). The cancer antigen (CA)-125 value was 19.68 U/ml. Other tumor markers like β human chorionic gonadotropin (hCG), alpha-fetoprotein (AFP), serum lactate dehydrogenase (LDH), and inhibin were normal. To confirm the nature of the lesion pre-operatively for planning optimal management, magnetic resonance imaging (MRI) of the abdomen and pelvis was performed, which revealed a lobulated heterogeneous solid-cystic lesion of size 7.1x8.1x12.0 cm with central cystic/necrotic degeneration on the right side of the pelvis, with mass effect compressing the uterus posteriorly and the urinary bladder inferiorly. There was a band of T2 hypointensity separating the lesion from the uterus on all planes, characteristic of an ovarian fibroma (Figure 1C). No obvious communication was seen with the uterus. However, bilateral ovaries were normally visualized and the rest of the abdominal organs were normal.

**Figure 2:**
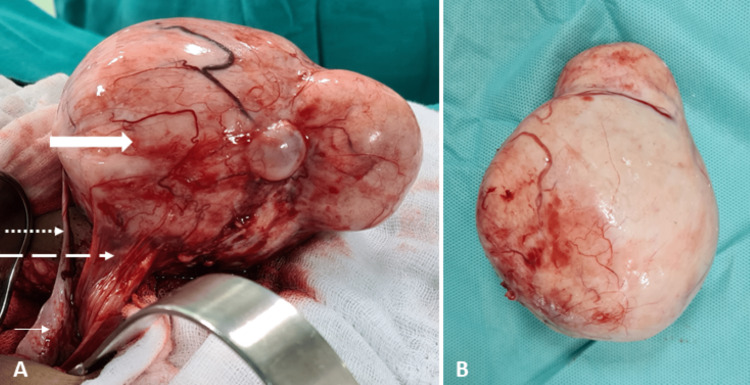
Operative images A. A large bilobed solid tumor (thick arrow) with peripheral vascularity attached to the right ovary (thin solid arrow) by a thin band (dotted arrow) and deriving blood supply from the bladder peritoneum (dashed arrow) suggestive of a parasitic ovarian tumor. B. The resected specimen of ovarian fibroma, measuring 12x8 cm.

In view of the diagnostic dilemma and the large size of the lesion, an exploratory laparotomy was planned and performed after discussion with the multidisciplinary team (MDT) with general surgery and onco-surgery. Intraoperatively, the uterus and bilateral ovaries were normal in size. A large, firm, bilobed solid tumor, tan-white in color with peripheral vascularity was present, which was adherent to the bladder peritoneum and attached to the right ovary by a thin band, suggestive of a right ovarian parasitic tumor (Figure 2A).

The mass was excised in toto and measured 12x8 cm (Figure 2B). Histopathology confirmed an ovarian fibroma.

## Discussion

The differential diagnosis of a solid and mobile adnexal tumor in a woman of reproductive age is a pedunculated subserous fibroid, ovarian tumor, and GIST [1,2]. Our patient was initially diagnosed on clinical examination as a case of pedunculated subserous fibroid. A uterine fibroid is the most common benign tumor of the uterus, usually affecting women in the third and fourth decades of life. Each fibroid is thought to be monoclonal in origin, arising from smooth muscle cells and fibroblasts of the uterine myometrium. However, a subserous pedunculated fibroid is uncommon and accounts for less than 0.25% of women with subserous uterine fibroids undergoing surgical intervention. On USG, a fibroid in the uterus appears as a hypoechoic lesion with the same echotexture as the myometrium. Larger fibroids (>5 cm) may show central anechoic spaces suggestive of degeneration. Pedunculated subserous fibroids show the characteristic ‘bridging vascular sign’ with the uterus, both on USG and MRI [4]. The ‘bridging vascular sign,’ caused by feeding vessels that arise from the uterine arteries, course through the myometrium, and supply large (>3 cm) exophytic myomas, indicates the uterine origin of a juxtauterine mass. In our case, the ‘bridging vascular sign’ was absent, hence the initial diagnosis of a subserous uterine myoma was ruled out.

Classification of adnexal lesions using the international ovarian tumor analysis (IOTA) group models helps in the preoperative assessment of the type, nature, and extent of the ovarian lesion [5]. This has the tumor marker CA-125 incorporated. Other tumor markers like hCG and AFP should be performed if the USG is suggestive of a germ cell tumor of the ovary. Serum LDH is performed if there is preoperative suspicion of leiomyosarcoma and inhibin if the USG is suggestive of granulosa cell tumor. In our case, these tumor markers were within the normal range. 

The presence of a large abdominal lesion with central degeneration in a woman with abdominal pain raised the possibility of GIST in our case. GIST is a common malignant lesion of the gastrointestinal tract arising from the interstitial cells of Cajal located within the muscle layer of the gastrointestinal tract. On USG, GIST usually appears as a hypoechoic solid mass with irregular borders, a heterogeneous echo pattern, and anechoic spaces [2]. In a five-year retrospective study of patients presenting with an abdominal/pelvic mass or pelvic pain, 10 patients were identified with GIST of either the small intestine, sigmoid colon, or base of small bowel mesentery. The mean tumor size was 13.9 cm [2]. However, as our index case did not have any of the other clinical symptoms of GIST like melena, hematemesis, and anemia, this was thought to be an unlikely diagnosis. It is important to differentiate these tumors preoperatively, as GIST is a malignancy, does not fall under the purview of gynecological surgery, and may require adjuvant treatment with tyrosine kinase inhibitors like imatinib. The salient differential points between an ovarian fibroma, subserous pedunculated fibroid, and GIST are listed in Table 1.

**Table 1:** Common differential diagnoses of ovarian fibroma USG: ultrasonography; EUS: endoscopic ultrasound; CT: computed tomography T1: longitudinal relaxation time; T2: transverse relaxation time; KIT: tyrosine kinase receptor; CD: cluster of differentiation

	Subserous pedunculated fibroid	Gastrointestinal stromal tumor	Ovarian fibroma
Prevalence	Less than 0.25% of women with subserous uterine fibroids undergoing surgical intervention	Most common malignant subepithelial lesions of the gastrointestinal tract	Most common sex-cord stromal tumor
Age	Third and fourth decades	Sixth decade	Fifth decade
Origin	Monoclonal from smooth muscle cells and fibroblasts of the uterine myometrium	Interstitial cells of Cajal located within the muscle layer of the gastrointestinal tract	Gonadal stromal cell origin from the ovarian cortex
Clinical features	Asymptomatic; acute pain if torsion ensues	Gastrointestinal bleeding, including acute melena and hematemesis with subsequent anemia; weakness; and abdominal pain, distension, and discomfort due to a tumor-induced mass effect	Usually asymptomatic, often detected at palpation during a routine gynecologic examination, can reach a large size at presentation. Associated with ascites (40%) and pleural effusion in a small percentage of cases
Imaging	USG: hypoechoic lesion with the same echotexture as the myometrium; color Doppler USG: Presence of bridging vascular sign MRI: hypo-intense homogeneous T2 signal and iso-intense T1 signal compared to myometrium; Necrobiotic leiomyomas have a heterogeneous hyper-intense T2 signal.	EUS: hypoechoic solid mass with a size of > 2 cm, irregular borders, heterogeneous echo patterns, anechoic spaces, echogenic foci, and growth during follow-up.	USG: solid, hypoechoic mass rarely with cystic component CT: diffuse, hypoattenuating mass MRI: well-circumscribed mass with low signal intensity; scattered hyperintense areas if cystic degeneration; a band of T2 hypointensity separating the tumor from the uterus on all imaging planes is a characteristic feature.
Pathology	On the cut surface: tan-white, and whorled with intersecting fascicles of monotonous spindle cells with indistinct borders, eosinophilic cytoplasm, cigar-shaped nuclei, and small nucleoli	KIT- or CD34-positive spindle-shaped cells or epithelial cells	Spindle cells with variable amounts of collagen, chalky-white surface with a whorled appearance on cut-section.
Management	Surgical resection or myomectomy	Surgical resection in localized disease, tyrosine kinase inhibitors like imatinib in metastatic disease	Surgical resection

An ovarian fibroma is an uncommon sex cord-stromal tumor and commonly affects women in the fifth decade of life [1]. However, in our case, this was diagnosed in the third decade. It originates from the gonadal stromal cells of the ovarian cortex and can reach a large size at presentation. In a large retrospective series, the most common presenting symptom of ovarian fibroma was abdominal pain and the mean size was 9.5 cm [1]. In our case too, it was a large tumor, 12x8 cm in size. On MRI, ovarian fibroma appears as a well-circumscribed mass with low signal intensity, with scattered hyperintense areas if cystic degeneration is present and a band of T2 hypointensity separating the tumor from the uterus on all imaging planes as a characteristic feature, as seen in our case also. However, in our case, bilateral ovaries were normally visualized by MRI, adding to the diagnostic dilemma.

 Still, the ovarian fibroma being parasitic was only discovered intra-operatively. Very few case reports of auto-amputation of ovarian fibromas with subsequent parasitic attachment have been previously reported [3]. In our case, this also explains the ‘wandering’ nature of the mass in the abdomen, as described by the patient. Pain may be felt due to the dragging effect of the large mass. Histopathology continues to be the gold standard of pathological diagnosis.

## Conclusions

To conclude, ovarian fibroma is a rare benign solid tumor of the ovary, and its being parasitic is even rarer. It may grow to a large size before becoming symptomatic. Misdiagnosis is common preoperatively, leading to a diagnostic dilemma. In our case, the patient complained of a 'wandering' mass in her abdomen. In retrospect, this was probably because the fibroma was attached to the ovary by a thin band only. The differential diagnosis in our case was a subserous pedunculated uterine fibroid, GIST, and ovarian tumor. A careful clinical approach and USG helped us rule out subserous pedunculated uterine fibroid, but the possibility of GIST was there. MRI pelvis showed a band of T2 hypointensity separating the lesion from the uterus on all planes, characteristic of ovarian fibroma. However, this being parasitic was discovered only during exploratory laparotomy. Surgery is curative.
